# An annotated dataset for extracting gene-melanoma relations from scientific literature

**DOI:** 10.1186/s13326-021-00251-3

**Published:** 2022-01-19

**Authors:** Roberto Zanoli, Alberto Lavelli, Theresa Löffler, Nicolas Andres Perez Gonzalez, Fabio Rinaldi

**Affiliations:** 1 Fondazione Bruno Kessler (FBK), Via Sommarive 18, Povo (TN), 38123 Italy; 2 Department of Neurology, Inselspital, Freiburgstrasse 16, Bern, 3010 Switzerland; 3 Department of Quantitative Biomedicine, University of Zürich, Winterthurerstrasse 190, Zürich, 8057 Switzerland; 4 Dalle Molle Institute for Artificial Intelligence (IDSIA USI/SUPSI) Campus Est, Via la Santa 1, Lugano, 6962 Switzerland; 5 Swiss Institute of Bioinformatics (SIB), Lausanne, 1015 Switzerland

**Keywords:** Melanoma, Annotated Dataset, Relation Extraction, Machine Learning, Deep Learning

## Abstract

**Background:**

Melanoma is one of the least common but the deadliest of skin cancers. This cancer begins when the genes of a cell suffer damage or fail, and identifying the genes involved in melanoma is crucial for understanding the melanoma tumorigenesis. Thousands of publications about human melanoma appear every year. However, while biological curation of data is costly and time-consuming, to date the application of machine learning for gene-melanoma relation extraction from text has been severely limited by the lack of annotated resources.

**Results:**

To overcome this lack of resources for melanoma, we have exploited the information of the Melanoma Gene Database (MGDB, a manually curated database of genes involved in human melanoma) to automatically build an annotated dataset of binary relations between gene and melanoma entities occurring in PubMed abstracts. The entities were automatically annotated by state-of-the-art text-mining tools. Their annotation includes both the mention text spans and normalized concept identifiers. The relations among the entities were annotated at concept- and mention-level. The concept-level annotation was produced using the information of the genes in MGDB to decide if a relation holds between a gene and melanoma concept in the whole abstract. The exploitability of this dataset was tested with both traditional machine learning, and neural network-based models like BERT. The models were then used to automatically extract gene-melanoma relations from the biomedical literature. Most of the current models use context-aware representations of the target entities to establish relations between them. To facilitate researchers in their experiments we generated a mention-level annotation in support to the concept-level annotation. The mention-level annotation was generated by automatically linking gene and melanoma mentions co-occurring within the sentences that in MGDB establish the association of the gene with melanoma.

**Conclusions:**

This paper presents a corpus containing gene-melanoma annotated relations. Additionally, it discusses experiments which show the usefulness of such a corpus for training a system capable of mining gene-melanoma relationships from the literature. Researchers can use the corpus to develop and compare their own models, and produce results which might be integrated with existing structured knowledge databases, which in turn might facilitate medical research.

## Background

The spread of melanoma has increased in the last 30 years [1]. The American Cancer Society estimates 96,480 new melanoma cases in 2019 in the United States, while 7,230 people are expected to die of melanoma [1]. Melanoma is a skin cancer that starts when the genes that control the cell division and reproduction are damaged [2]. This causes the cell to divide and grow in number without control. Understanding the genes involved in melanoma is thus essential for progress in the accurate diagnosis and in therapeutic results for patients with melanoma.

**Fig. 1:**
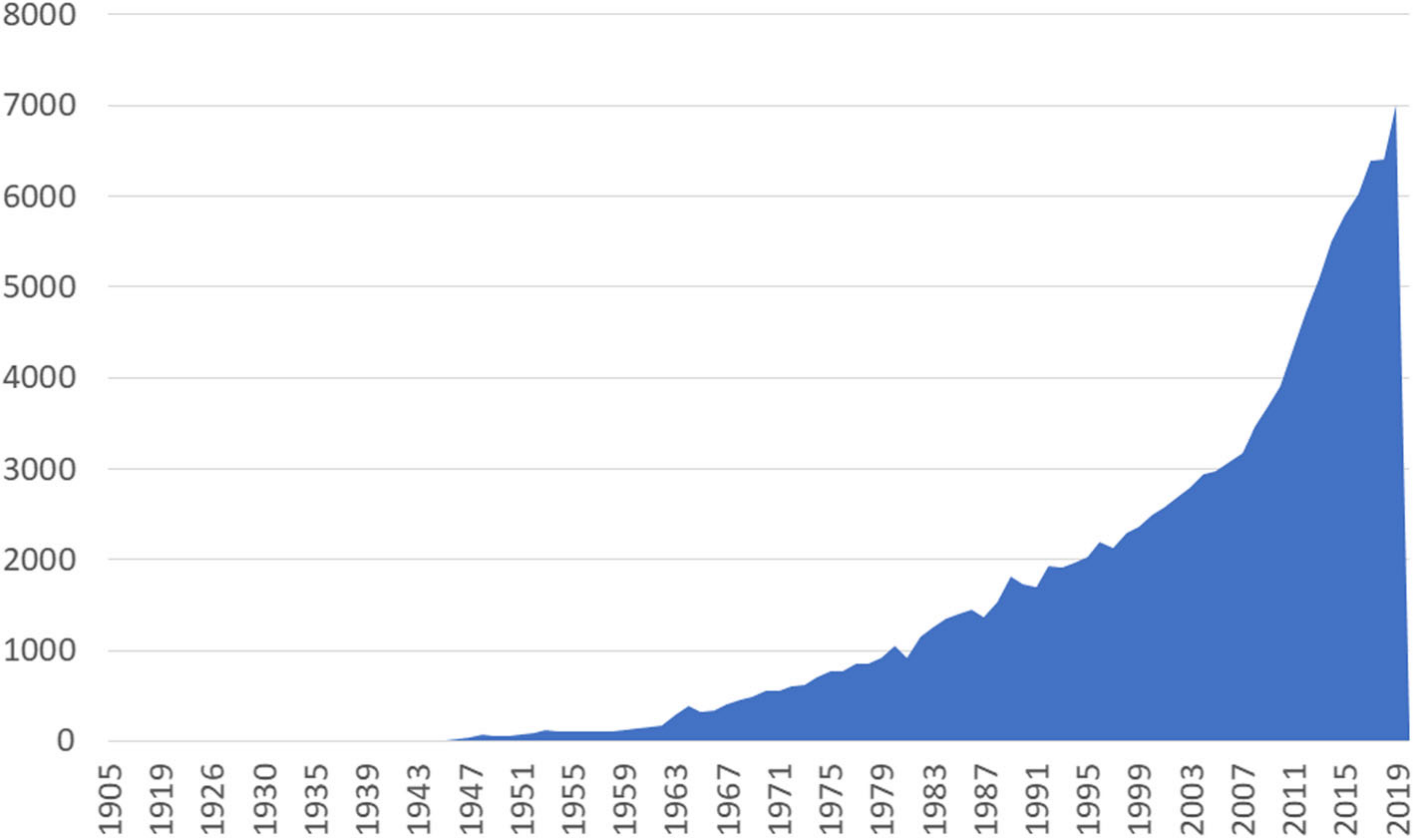
Number of publications on melanoma disease per year in PubMed

Many genes related to human melanoma have been studied, and many publications reporting new genes associated with prognosis in melanoma are being published every year (Fig. 1). Since the publications have no pre-defined format or organization, the retrieval of these genes can only be conducted on the basis of their co-occurrence with melanoma within a publication. Unfortunately, genes and melanoma diseases can be mentioned together without any causal relation between them. This forces researchers to analyze a large amount of documents to find the actual relation of interest.

To provide researchers with formats that can be more easily queried and analyzed, gene-melanoma relations in the publications must first be annotated. This can be done in two ways: (i) Biological curation of data produces accurate annotations. However, it is costly and difficult to keep up-to-date. (ii) Machine learning-based approaches for automatic relation extraction are a faster and cost-effective alternative to data curation. Nevertheless, the use of machine learning has been extremely limited due to the lack of annotated resources.

In order to apply machine learning to bio-entity relation extraction, we have generated a freely available dataset called **MelanoBase Gene Relation (MGR) base dataset.** This dataset consists of 907 publications about melanoma extracted from PubMed. The publications are annotated at two levels: (i) entities of type gene and melanoma, and (ii) binary relations between genes and melanoma entities.

Both the entities and the relations in our dataset were automatically annotated by exploiting the information of the Melanoma Gene Database (MGDB), a manually curated catalog of human melanoma related genes. In MGDB the relationships between genes and melanoma were manually curated from PubMed abstracts. For each gene the database reports the PubMed identifiers of the publication abstracts where the causal relation is expressed. Then, for each abstract MGDB contains the text snippet, i.e., a portion of text that provides evidence supporting the existence of the relation. For example, for gene 〈Apaf-1 〉 in abstract 〈PubMed ID: 15305193 〉 the following snippet has been extracted:

*our data indicate that Apaf-1 expression is significantly reduced in human melanoma*.

We used state-of-the-art text-mining tools to annotate the entities in the abstracts. This annotation includes both the mention text spans (e.g., Apaf-1) and the normalized concepts identifiers (e.g., 317). For example, in our dataset the snippet above is annotated as follows:

*our data indicate that* [***Apaf-1***]_317_ *expression is significantly reduced in human* [***melanoma***]_D008545_.

The relations between the entities were annotated at concept- and mention-level. The concept-level annotation was produced using the information of the genes in MGDB to establish a relation between the recognized gene and melanoma concepts in the annotated abstract. Given the dependency of relations on concepts, our test collection contains the gene/melanoma annotations, the full text abstracts retrieved from PubMed, and the relation annotations in the same set of articles. The exploitability of this annotation was tested both with traditional machine learning models, and with more recent neural network-based models. The resulting models were applied to annotate a larger dataset (referred to as **MGR extended dataset** in the rest of the paper) of 89,137 publications from PubMed.

Relation extraction can be cast as a classification problem. Given a piece of text (generally a sentence rather than the entire abstract) that contains two entity mentions, current machine learning methods (e.g., [3, 4]) exploit the surrounding words of the target entity mentions to train a classifier. However, the concept-level annotation of MGDB is not directly applicable to these methods and must be transformed to mention-level instances before being used by the classifiers. MGDB contains the snippet of the related mentions, but it does not specify the exact position of the target entities in the snippet that is required to apply the machine learning approaches mentioned above.

To deal with this issue, Distant Supervision methods for relation extraction [5] have been considered. These methods rely on the assumption that, if two entities are involved in a relation, any sentence that contains these two entities would express such relation. Therefore, erroneous relation instances can be produced. However, it is expected that the majority of the annotated instances will be correct, thus creating a large number of correctly labeled instances against a small number of incorrectly labeled instances. This kind of annotated corpus, although less ideal than one generated by manual annotation, is still usable by machine learning algorithms, and the cost of creating it is much smaller.

The MGR base dataset extends the information of MGDB through the annotation of such missing target entities. Researchers will be able to use the concept-level annotation of the MGR base dataset to develop their predictive models. Moreover, the relations extracted from the literature and included in the MGR extended dataset can be integrated with existing structured knowledge to help find new ways to diagnose and treat the melanoma. Finally, the mention-level annotation of the dataset is in support to researchers who want to test their models right away, exploiting the pre-processed entities and relations in the snippets of MGDB.

The “Related work” section briefly surveys related work. The “Methods” section describes the procedure followed to build the MGR base and MGR extended datasets. The “Results” section shows some statistics on the dataset and outlines the results obtained by machine learning models. Finally, the “Discussion” section presents and discusses our results.

## Related work

Most work in the field of text processing and melanoma focused on biological curation of data. In 2007 the Melanoma Molecular Map Project [6] was created as an open-access website dedicated to the systematic collection of scientific information on melanoma biology and treatment. MelanomaDB [7] is a web interface to a large database of melanoma genomic information. MelGene [8] is a database of published genetic association studies of Cutaneous Melanoma. In 2015, the Melanoma Gene Database [9] manually collected several gene-melanoma relationships from the biomedical literature.

In many areas of biology machine learning methods have been successfully applied to extract information from scientific literature. Initial experiments on relation extraction between biomedical entities were conducted in [3, 10]. More recently, deep learning techniques, such as Convolutional Neural Network (CNN) and Recurrent Neural Network (RNN), have proved to achieve substantially better results than more classical models in various Natural Language Processing (NLP) tasks, including relation extraction [11–13]. These models require large amounts of annotated training data that are difficult to obtain. Hence, many methods have been developed to take advantage of unannotated data, such as transfer learning and fine-tuning of pre-trained models [14]. In NLP, typical pre-training approaches include word vector models like Word2Vec [15] and Bidirectional Encoder Representations from Transformers (BERT) [16].

A considerable number of challenges have been organized so far with the aim of providing annotated datasets that can be used by researchers to develop and compare their approaches. In 2013, SemEval-2013 Task 9 [17] concerned the extraction of drug-drug interactions from biomedical literature. In 2015 BioCreative V Track 3 [18] focused on chemical-disease interactions, and in 2017 BioCreative VI Track 5 [19] was related to chemical-protein interaction. In 2019, the AGAC track of BioNLP Open Shared Tasks [20] consisted of three subtasks: 1) named entity recognition, 2) thematic relation extraction, and 3) loss of function (LOF) / gain of function (GOF) topic classification.

As regards text mining tools for melanoma research, MelanomaMine^1^ is a machine learning application for recognizing bio-entities like genes, and chemicals of relevance to address the molecular basis of melanoma. However, the automatic extraction of relations between the extracted entities and melanoma has remained unexplored. iTextMine [21] is an application that integrates four relation extraction tools for large-scale knowledge extraction from the biomedical literature. In addition to detecting association between genomic anomaly and drug responses, this application also records the associated drug and disease.

Finally, it is worth mentioning a recent work done by Lever et al. [22] which has some shallow similarities to ours. They created a dataset of 1,500 manually annotated sentences that discuss genes related to cancer. Then, they used this dataset to train machine learning models to extract 35,951 relations from 26,767 PubMed publications. In their manually annotated dataset we counted 186 different types of cancer including the melanoma cancer. However, only 96 out of 1,500 sentences concern melanoma. Our annotated dataset originates from a much larger number of text sentences focusing on genes associated to melanoma (1,403 vs 96). For this reason, we believe it may well produce more accurate models to predict new potential gene-melanoma associations.

## Methods

In this section, we briefly review the core content of MGDB used to build the MGR base dataset. Then, the procedure to collect the genes linked to melanoma is described. Next, we show how the dataset was exploited with machine learning techniques. Finally, we report on the procedure to annotate the large collection of publications about melanoma that are part of the MGR extended dataset.

### MGDB

**Fig. 2:**
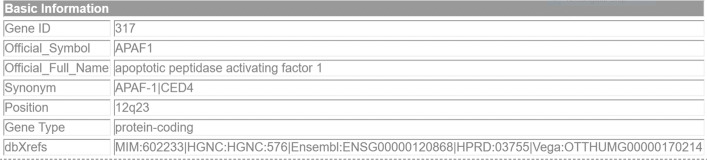
The MGDB Basic Information page reports a description of the annotated genes (in this case APAF-1)

In MGDB (Melanoma Gene Database), the genes and their relationships with melanoma were manually extracted from PubMed abstracts. Each gene in the database can be accessed through the gene Basic Information web page (Fig. 2).

**Fig. 3:**
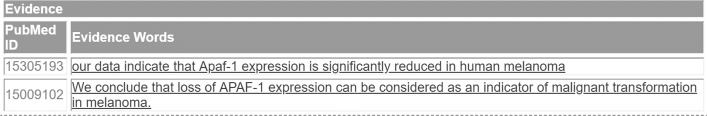
The snippets associated to a gene (in this case APAF-1) contain the text-evidence to support the relation between the gene and melanoma

This page contains the GeneID (e.g., 317) in Entrez Gene [23], the gene official symbols (e.g., APAF-1), and the synonyms of the gene in Entrez Gene (e.g., CED4). Besides, the Basic Information page includes the PubMed ID (PMID) of the abstract, and the snippets that explicitly support the causal relation between the gene and melanoma (Fig. 3) in the abstract. However, the full text abstract is absent.

A snippet can be a complete sentence in the abstract, but also a smaller fragment of it, or it can be split across adjacent sentences. An example for each of the three cases is given below:

Complete sentence: *BRAF and NRAS mutations are commonly acquired during melanoma progression.*

Smaller fragment: *The results confirm that c-kit is vastly expressed in uveal melanoma,...*

Across adjacent sentences: *Previously identified as well as novel driver genes were detected by scanning CNAs of breast cancer, melanoma and liver carcinoma. Three predicted driver genes (CDKN2A, AKT1, RNF139) were found common in these three cancers by comparative analysis.*

The genes in the snippets can be mentioned by their official symbol, by their full name, by one of their synonyms, but also by a pronoun. Melanoma can appear in any of its variants depending on whether it affects the skin (e.g., skin melanoma), the eyes (e.g., uveal melanoma), or for example the nasal mucosa (e.g., mucosal melanoma). Melanoma is also mentioned using generic terms such as tumor or cancer or even, in a few cases, there can be no direct mention as in the following example:

*Importantly, metastatic outgrowth was found to be consistently associated with activation of the transforming growth factor-beta signaling pathway (confirmed by phospho-SMAD2 staining) and concerted up-regulation of POSTN, FN1, COL-I, and VCAN genes-all inducible by transforming growth factor-beta).*

Table 1 summarizes the data on MGDB at two levels of analysis granularity.

**Table 1:** MGDB contains 1,272 relations at concept-level. ≥1,403 at mention-level

Genes	PMID	Snippets	Relations	
			concept level	mention level
527	910	1,403	1,272	≥1,403

**Fig. 4:**
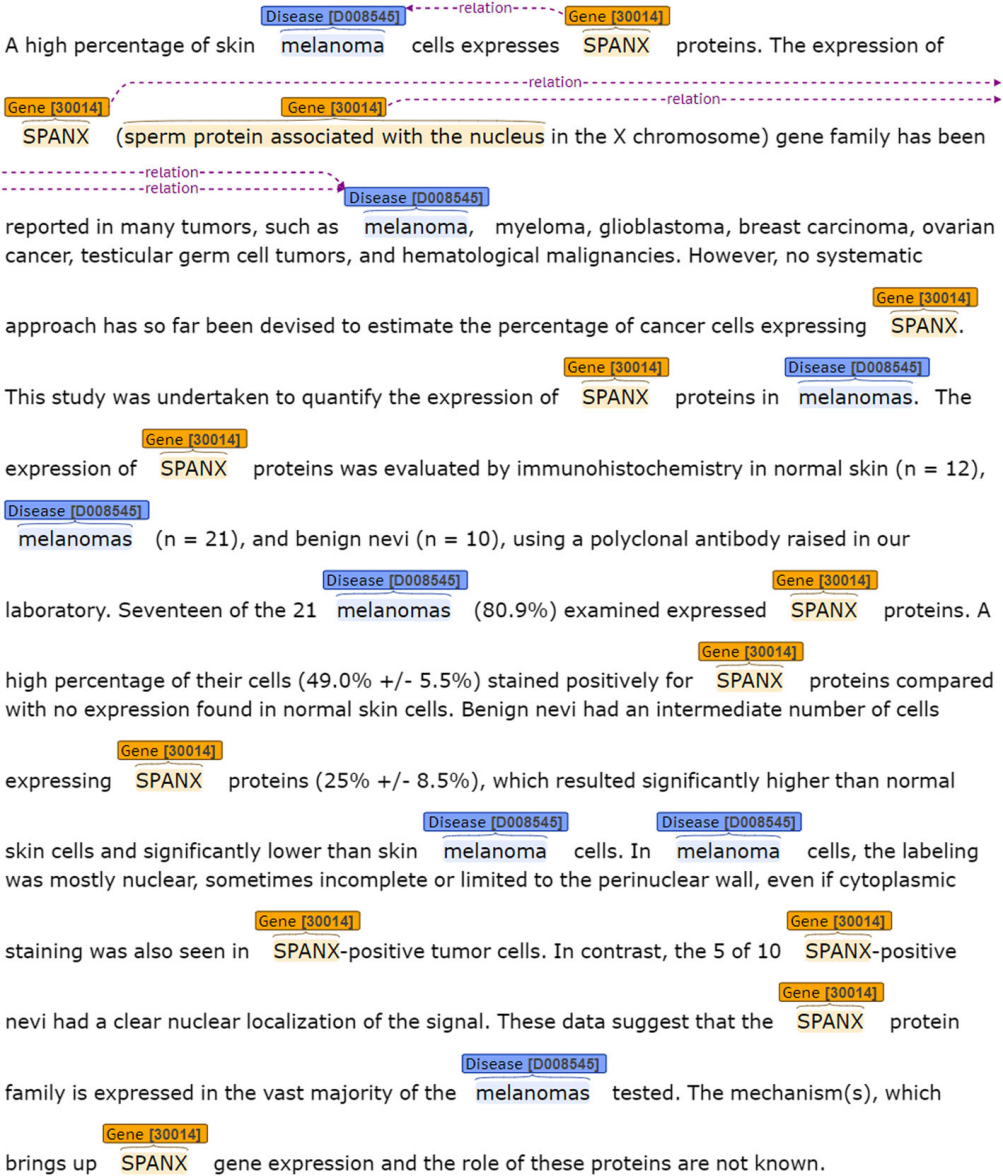
Concept-level: there is one relation between gene 〈ID: 30014 〉 and melanoma 〈ID: D008545 〉. Mention-level: there are three relations between three mentions (SPANX, SPANX, sperm protein associated with the nucleus) of gene 〈ID: 30014 〉 and two mentions (melanoma, melanoma) of disease 〈ID: D008545 〉

At the level of concepts, where an entity consists of all the mentions which refer to one conceptual entity, the database contains 1,272 relations between the gene and melanoma concepts that co-occur in 910 abstracts. At the level of mentions, where the same snippet could contain more than one mention of the gene and where the exact position of the mentions is unknown, the number of relations can only be estimated based on the number of snippets. Since one snippet contains at least one relation, we can assume that 1,403 relations is a reasonable conservative estimate of the total number of relations for the 1,403 snippets. Figure 4 provides a concrete example for the two types of concept- and mention-level annotation.

In article 〈PMID: 19318807 〉 gene 〈SPAN-X 〉 has thirteen mentions formed by the set {SPANX, sperm protein associated with the nucleus} but one concept (ID: 30014). Melanoma has eight mentions formed by the set {melanoma, melanomas} but one concept (ID: D008545). At concept-level there is one relation between gene 〈ID: 30014 〉 and melanoma 〈ID: D008545 〉. At mention-level there are three relations. The first relation is between one mention (SPANX) of concept 〈ID: 30014 〉 and one mention (melanoma) of concept 〈ID: D008545 〉 expressed in the snippet:

*A high percentage of skin melanoma cells expresses SPANX proteins.*

The second relation is between one mention (SPANX) of concept 〈ID: 30014 〉 and one mention (melanoma) of concept 〈ID: D008545 〉. Then, the third relation is between one mention (sperm protein associated with the nucleus) of concept 〈ID: 30014 〉 and one mention (melanoma) of concept 〈ID: D008545 〉. These last two relations are expressed in the snippet:

*The expression of SPANX (sperm protein associated with the nucleus in the X chromosome) gene family has been reported in many tumors, such as melanoma,*

Despite the high quality of the annotated relations in MGDB, this annotation suffers from some limitations that need to be addressed to make it consumable by text mining algorithms. These limitations can be summarized as follows:

- Relations between two entities can appear in many snippets of an abstract, but sometimes MGDB just reports one snippet for each single entity pair in the abstract. Browsing the database we observed a certain number of such cases. For example, for document 〈PMID: 23537197 〉 and gene 〈PARP1 〉 the following snippet has been annotated in MGDB:
  *Genetic variants in PARP1 (rs3219090) and IRF4 (rs12203592) genes associated with melanoma susceptibility in a Spanish population.*
  Nevertheless, such document contains other snippets where the same relation is expressed, e.g.,
  *We confirm the protective role in Malignant Melanoma of the rs3219090 located on the PARP1 gene (p-value 0.027).*
  *We confirmed the proposed role of rs3219090, located on the PARP1 gene, and rs12203592, located on the IRF4 gene, as protective to Malignant Melanoma.*
- The position of the genes and melanoma in the snippets is unknown. Moreover, a gene and melanoma can occur more than once in a snippet. For example, to provide evidence of the relation between gene EIF1AX and melanoma in article 24423917, MGDB shows this snippet:
  *The tumour showed mutations in GNA11 and EIF1AX that are typical for uveal melanoma and absent from cutaneous melanoma.*
- Melanoma entities are not always explicitly mentioned in the snippets. For example, for article 15133496 and gene FAP, this snippet is reported:
  *FAP expression is specifically silenced in proliferating melanocytic cells during malignant transformation.*
- There is no information about the type of melanoma induced by the genes.

### MGR base dataset collection

To overcome the limitations described at the end of the previous section and build the MGR base dataset, the following 5 steps were applied.

1. *Genes collection* from MGDB was performed by querying the genes Basic Information web page (Fig. 2). For each gene, we got its GeneID, its snippets and the PMID of the abstracts containing the snippets (Fig. 3). These data were downloaded on March 27, 2019 from the MGDB website^2^.
2. *Abstracts collection* was conducted using the PMID from step 1 as input to retrieve the abstracts from PubMed^3^. These data were collected on April 4, 2018. Two are the main reasons why we used the whole abstracts instead of using the individual snippets. Firstly, a snippet in MGDB does not always match with a complete sentence. For example, for document 〈PMID: 23103111 〉 and gene 〈OCA2 〉 this snippet has been annotated:
  *supports existing GWAS data on the relevance of the OCA2 gene in melanoma predisposition,*
  However, NLP tools often require entire sentences to work properly. Secondly, the annotated entities in the abstracts that do not take part in any relationship were used to generate the negative examples necessary for applying machine learning techniques.
3. *Mention detection* was obtained with OGER [24], a fast and accurate Named Entity Recognition and Linking tool for the biomedical domain. Firstly, the gene and disease mentions in the abstracts were recognized. OGER was configured to annotate the entities that are included in Entrez Gene. We annotated their text span (e.g., RUNX3, malignant melanoma), and their category (i.e., Gene, Disease). Secondly, the human genes were selected by matching the gene mentions with the human genes in MIM (Mendelian Inheritance in Man) [25]. Finally, the melanoma diseases were picked out by retaining those disease mentions whose text span contained the term *melanoma*. Coreference resolution of terms such as *tumor* or *cancer* to recognize a small minority of remaining and not yet recognized melanoma diseases is left to a subsequent study.
4. *Concept identification* was applied to (i) annotate the genes recognized in the previous step with their concept identifier from Entrez Gene produced with OGER, (ii) associate the most general concept of *melanoma* (ID: D008545) in the MeSH hierarchy to the annotated diseases. The fact that MGDB does not specify any information about the type of melanoma involved in the relation is the reason for adopting a unique concept ID for all the melanomas.
5. *Relation extraction* between the annotated mentions and concepts was performed by leveraging the mention- and concept-level annotation provided with MGDB.
  The mention-level annotation of the dataset was created by linking the gene and melanoma mentions co-occurring within a snippet on condition that (i) such relation exists in MGDB, and (ii) the entity mentions in the snippet are not split across sentences. Since a snippet can contain multiple mentions of the same gene or melanoma concept, and MGDB does not specify the mention pair involved in the relationship, this can lead to the production of multiple gene-melanoma relations. For example, in the snippet below three mentions of gene 〈STK11 〉 (LKB1, STK11, **LKB1**) are linked to melanoma by our procedure, even if only one of such mentions (**LKB1**) is actually involved in the relationship.
  *Germline mutations in the LKB1 gene (also known as STK11) cause the Peutz-Jeghers Syndrome, and somatic loss of* ***LKB1*** *has emerged as causal event in a wide range of human malignancies, including* ***melanoma***, *lung cancer, and cervical cancer.*
  We estimate that 14,87% of the snippets in MGDB contain more than one mention of the same entity, and that therefore, they could cause the issue. However, this percentage also includes several cases where indeed multiple mentions of a gene all participate in the relationship. For example, in the snippet below both 〈bone morphogenetic protein 4 〉 (gene official full name) and 〈BMP4 〉 (gene official symbol) are in relationship with melanoma:
  *An altered expression of bone morphogenetic protein 4 (BMP4) has been found in malignant melanoma cells.*
  Another example similar to the one above is the following. In the following snippet both 〈melanocortin 1 receptor 〉 (gene official full name) and 〈MC1R 〉 (gene official symbol) are involved in the relationship:
  *Furthermore variants in melanocortin 1 receptor (MC1R) and microphthalmia-associated transcription factor (MITF) give a moderately increased risk to develop melanoma.*
  Generating the mention-level annotation aims at (i) preserving the snippets that in MGDB express the relations, (ii) providing researchers with automatically pre-processed entities mentions. The SemEval-2013 Task 9 dataset is an example of a dataset that uses a similar type of annotation.
  The concept-level annotation was produced generating one relation for each 〈gene,melanoma 〉 concept pair in an abstract, provided that such relation exists between two mentions of these concepts in a snippet of the abstract. It offers a natural way to (i) go beyond the limitation of the annotation used in MGDB, which sometimes reports just one snippet per relation, (ii) exploit the information at mention-level to decide if a relation holds between two concepts in the abstract. Examples of datasets that use a similar kind of annotation are the BioCreative V Track 3 and BioCreative VI Track 5 datasets.

At the end of these steps, there are 211 out of 1,403 (15.04%) relations at mention-level and 28 out of 1,272 (2.2%) at concept-level which, despite being in MGDB, have not been annotated in the dataset.

Regarding the mention-level annotation, we manually verified that most of the missed relations concern occurrences of melanoma which are not explicitly mentioned in the snippets, and therefore we cannot recognize (140 mentions); the other cases refer to genes not correctly identified by OGER (71 mentions). Below, we report an example of each of the two cases:

Melanoma not explicitly mentioned: *BRAF and GNAQ mutations in melanocytic tumors of the oral cavity.*

Entities not recognized by OGER (e.g., miR-222): *this suggests that targeted therapies suppressing miR-221/-222 may prove beneficial in advanced melanoma.*

As far as the concept-level annotations are concerned, the missing relations are due to genes not correctly identified (26 concepts), and melanoma entities that we could not recognize (3 concepts). Table 2 shows that, despite these shortcomings, more than 84% of the relations in MGDB have been maintained in the mention-level dataset and more than 97% in the concept-level dataset. Eventually, 3 out of 910 abstracts were found to be without relations and were removed from consideration.

**Table 2:** Number of relations in MGDB and of relations that have been maintained in the MGR base dataset

MGDB		MGR base dataset	
concept level	mention level	concept level	mention level
1,272	1,403	1,244(97.80%)	1,192(84.96%)

**Fig. 5:**
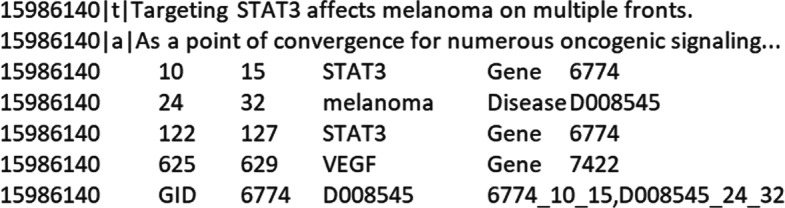
PubTator annotation for article 〈PMID: 15986140 〉. Concept-level: gene 〈ID: 6774 〉 is related to melanoma 〈ID: D008545 〉. Mention-level: gene 〈ID: 6774 〉 at position START:10,END:15 is related to melanoma 〈ID: D008545 〉 at position START:24,END:32

To ensure the reproducibility of our results the dataset is split (randomly) in two parts: a training set for training the models (2/3 of the data) and a test set (1/3) for the evaluation of the models. Figure 5 shows the annotation for article 〈PMID: 15986140 〉 in the test set. Coherently with the license adopted for MGDB, we make the dataset freely available^4^ for research purposes only.

### MGR base dataset validation

NLP annotation and biomedical curation applications are based on different needs. In NLP, relation extraction usually requires considering the mentions of given entities in the document, and to decide whether two specific mentions are connected by a relation. Conversely, typical curation applications only need to know that a document supports the existence of a relation between two given entities (without considering the specific mentions expressing the relation).

With this view in mind, we formulate the relation extraction task as a two-class classification problem. First, in the training phase our models are trained on the mention-level annotation of the dataset that specifies the mention pair involved in the relationship. Then, in the evaluation phase the trained models are used to predict whether in a sentence a given candidate mention pair is in relationship or not. Since the predicted relations have to be evaluated between pairs of entities rather than between pairs of mentions, the mention-level annotation produced by the models is transformed into the corresponding concept-level annotation. This is done assuming that a relationship between two entities exists if at least one relation between a mention pair of the entities is found by the models in the abstract.

Training the models at mention-level enables us (i) to apply standard NLP relation extraction approaches that work at sentence-level, rather than at document-level, (ii) to use the snippets reported in MGDB that explicitly support the given relations and that have been annotated with the target mentions. Evaluating the models at concept-level is in the spirit of better matching the actual requirements of practical applications.

Regarding the examples for training and testing the models, they were generated as follows. First, the dataset was processed with spaCy^5^ (i.e., model en_core_web_sm-2.0.0) for tokenization, sentence splitting, and lemmatization. Then, positive and negative examples were generated for all the sentences containing at least one gene and one melanoma entity. Concerning the training data, the following rule was adopted:


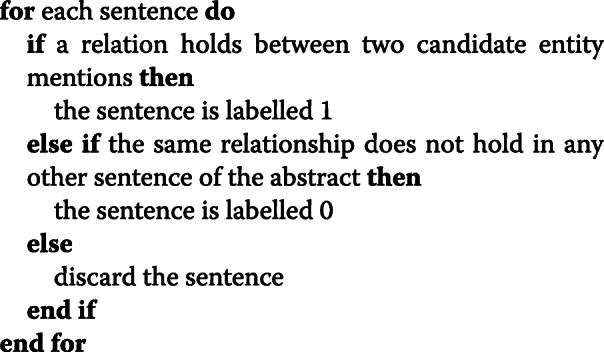


In the pseudo-code above, a relation holds if the sentence containing the two entity mentions is equal to or includes the text snippet that in MGDB captures the relation between the two mentions. The rationale of discarding some examples is to reduce false negative examples produced by models that were trained on relations that, despite being positive, were not annotated in the MGR base training set because of the *one snippet per relation* constraint.

We also create a “test dataset”, representative of the unlabeled data that would be found in a real world application of our system. In such test dataset each sentence represents an example to be classified. To evaluate the predicted relations between pairs of entities rather than between pairs of mentions, we also produced a concept-level annotation of the test dataset. In this regard, the following rule was adopted:


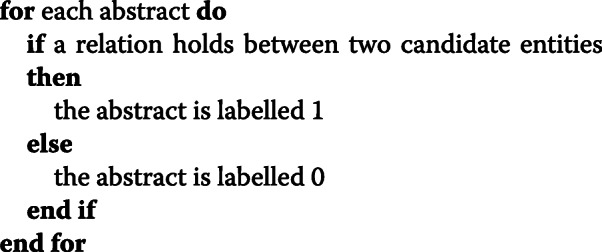


In the pseudo-code above, a relation holds if the abstract containing the two entities is equal to the abstract that in MGDB captures the relation between the two entities.

After data has been generated, each sentence was represented by a set of features that depends on the model used (see “Models” section).

For the models development the training set was split into two parts: a dev-train for training (2/3 of the training data mentioned in the previous section) and a dev-test for tuning the models (1/3). Then, the resulting models were used to annotate the test set.

Eventually, to establish a lower bound of expected performance on the dataset, we adopted the two baselines that were used at BioCreative V chemical-disease relation (CDR) task in the evaluation process: co-occurrence of genes and melanoma entities across sentences in the whole abstract (abstract-level baseline), and co-occurrence of genes and melanoma entity mentions in the same sentence only (sentence-level baseline).

### Models

The MGR base dataset was tested with both traditional models like *decision trees*, and more recent neural network-based models such as *Convolutional Neural Network* (CNN) and *Bidirectional Encoder Representations from Transformers* (BERT).

The success of deep learning for NLP applications largely depends on word vector representations, also known as word embeddings. Word embedding is one of the most popular word representations. With word embedding, words are represented by real-valued vectors of tens or hundreds of dimensions. This representation is learnt in such a way that words that are used in similar ways have similar vector representations. This is different from the thousands or millions of dimensions needed by more traditional word models, where each word has a unique representation, independent from how it is used. As an example, consider the two sentences: *POT1 gene has been associated with melanoma* and *POT1 gene has been implicated in melanoma*. Using pre-trained word embeddings, the neural networks used in our experiments can exploit the semantic relationships between *associated* and *implicated* (their vector representations are relatively close in vector space) to compute a distance between the two sentences. The same cannot be said for the decision-tree models that rely on traditional word representations. These models can only check whether a particular word, represented as a feature, exists or not. Early word embedding models, such as Word2Vec [15] used with our CNN approach, learn one fixed representation per word. However, this cannot capture how the meaning of a word depends on its context. Recently, systems like BERT [16] showed that generating a different embedding for each word in the context where it appears, can outperform previous methods. The methods that we have used for the experiments described in this paper are the following.

The **decision-tree** method implemented in scikit-learn^6^ is an optimized version of the Classification and Regression Trees (CART) algorithm. As features, we considered the lemma of the tokens in a window from two tokens before the leftmost entity of the pair, to two tokens after the rightmost entity of the pair.

The **CNN** approach is essentially the same as that described in [4]. The only change in our work is that we used pre-trained embeddings^7^ calculated on PubMed [26] instead of the ones calculated on more general-purpose text corpus. For our experiments, we used the code implementation of [27], in which each word is represented by concatenating its word-embeddings and the shortest distances relatively to the two entities in relationship.

**BioBERT** [28] is a version of BERT that has been pre-trained on large-scale biomedical corpora. BERT authors have shown that unsupervised pre-training of language models on a large corpus, followed by fine-tuning, is beneficial for many NLP tasks. BioBERT extends this work and demonstrates that pre-training BERT on additional biomedical corpora helps it to analyze complex biomedical texts. We conducted our experiments using the code and the default configuration provided with BioBERT [29]. As regards the input, we fed BioBERT with a plain text file that consists of one sentence for each positive and negative example.

### MGR extended dataset annotation

**Fig. 6:**
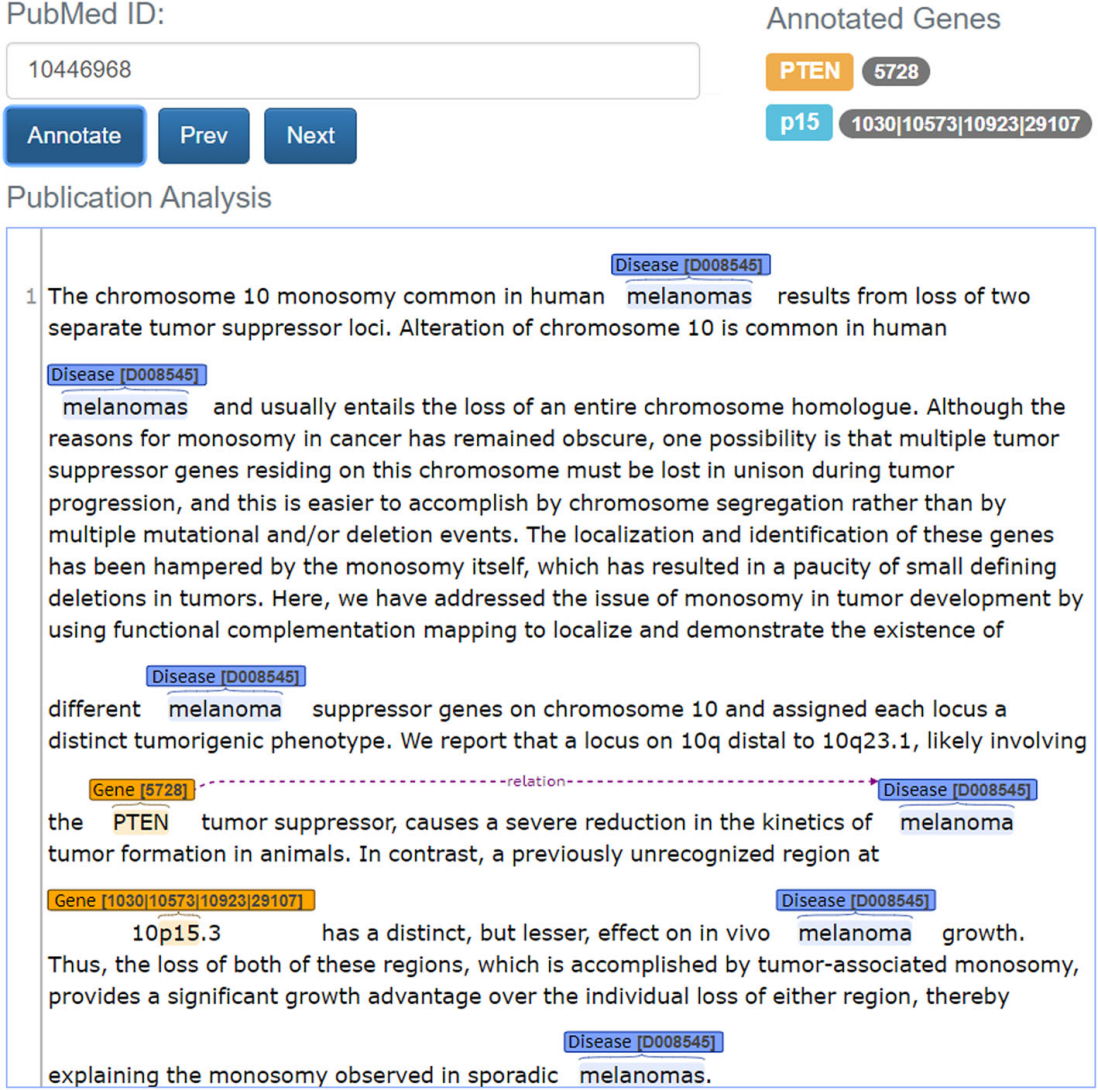
Extracted relation between gene 〈ID: 5728 〉 and melanoma 〈ID: D008545 〉 for article 〈PMID: 10446968 〉

To demonstrate the usefulness of the models implemented in the previous section, we used BioBERT fine-tuned on the mention-level MGR base training dataset to annotate a large set of PubMed abstracts about melanoma. First, the query in Additional file 1 was performed to get 90,028 publications abstracts. This query was run on April 4, 2018. Then, from these abstracts we removed the ones that are already annotated in MGDB. In this way we obtained 89,137 abstracts. After that, we followed the procedure described in points 3 and 4 of “MGR base dataset collection” section to annotate the gene and melanoma concepts in the abstracts. Finally, the relations among the annotated concepts were extracted with the BioBERT model. Figure 6 shows that in article 〈PMID: 10446968 〉 gene 〈ID: 5728 〉 is associated to melanoma 〈ID: D008545 〉. This dataset will be referred to as MGR extended dataset. We make it available to the research community in the Mendeley Data repository^8^.

A manual assessment of the quality of the entire MGR extended dataset would be too demanding in terms of human resources. For this reason, the quality of the dataset has been estimated through (i) a direct evaluation of a sample subset of relations (ii) an indirect evaluation using the results obtained on the MGR base dataset. Regarding the direct evaluation, two domain experts assessed the quality of a sample subset of relations. First, the relations in the MGR extended dataset were sorted by their decreasing scores produced by BioBERT. Then, from these relations we considered the relations involving genes not classified in MGDB. These relations should be the most difficult to extract for our classifier (they are not present in our training dataset), but also the most interesting because they are new and not in MGDB. As the output of this phase, we obtained 2,265 genes and 6,866 relations annotated at concept-level. Among these relations, the two domain experts were asked to verify the first 700 relations (those with the highest probability). The first expert manually checked the odd-numbered relations, while the second expert checked the even-numbered relations. At concept-level, 600 relations out of 700 were judged as correct (precision: 85.71), while at mention-level there were 1,070 out of 1,506 correct relations (precision: 71.05). The agreement between the two experts was calculated using the measure studied by Scott [30]. It is defined as the percentage of judgments on which the two experts agree when annotating the same data independently. To compute this measure, 100 additional relations were selected from the dataset and then verified by both the experts. At concept- and mention-level the observed agreement is 73.00% and 71.31% respectively. Concerning the indirect evaluation, we used the results obtained by the models on the MGR base dataset as a good estimator of the quality of the relations extracted from the MGR extended dataset. This approximation is possible because both the vast majority of publications in the MGR base dataset (precisely 891 out of 907) and all the publications in the MGR extended dataset belong to the same set of 90,028 abstracts extracted from PubMed. This allows us to consider with confidence the publications in the MGR dataset as representative of the publications in the MGR extended dataset.

## Results

Table 3 shows that the number of relations annotated at concept- and mention-level in the MGR base dataset (1,244 and 1,192 respectively) is roughly of the same order of magnitude as in other frequently used datasets for relation extraction. For example, the BioCreative V Track 3 dataset contains 3,116 relations at concept-level, while the SemEval-2013 Task 9 dataset consists of 5,021 relations at mention-level.

**Table 3:** Number of mentions (concepts) and relations for the MGR base training and test set splits

PMID	Mentions(ID)		Relations	
	gene	disease	concept level	mention level
train(605)	8,622(868)	4,251(1)	828	1076
test(302)	4,262(537)	2,073(1)	416	537
all(907)	12,884(1,127)	6,324(1)	1,244	1,613

Our models trained on the mention-level annotation of the dataset and evaluated at concept-level outperform the two baselines in terms of *F*_1_ measure (Table 4). The results of the models are also higher than the results of the best systems at BioCreative V Track 3 (*F*_1_: 57.03) and BioCreative VI Track 5 (*F*_1_: 64.10). The BioBERT results are as good as the results obtained by the BioBERT authors on the BioCreative VI Track 5 dataset (76.46).

**Table 4:** Precision (Pr), Recall (Re), and *F*_1_ measure of the models (BioBERT, CNN, decision tree) and baselines (sentence- and abstract-level) calculated on the concept-level test set

Models	Pr	Re	*F*_1_
BioBERT	74.42	77.29(67.09)	75.83
CNN	70.50	71.01(59.15)	70.76
decision tree	66.27	67.87(44.18)	67.06
sentence-level	49.68	94.93	65.23
abstract-level	35.75	100.00	52.67

In brackets the recall calculated on the mention-level test set

The percentage of relations present in the mention-level annotation of the dataset that are found by BioBERT and CNN (Table 4, recall values in brackets) is in the average of recall values obtained by the best system at SemEval-2013 Task 9 on the MedLine dataset (51%) and DrugBank dataset (84%).

The BioBERT model trained on the concept-level of the MGR base dataset took 104 minutes (NVIDIA Tesla K80 GPU 24GB) to extract 16,215 (2,657 distinct) relations from the 89,137 PubMed abstracts that are included in the MGR extended dataset (Table 5). Among the extracted relations we have found 2,265 new potential gene-melanoma relations that are not included in MGDB.

**Table 5:** Number of mentions (concepts) and relations in the MGR extended dataset

PMID	Mentions(ID)		Relations	Time [min]
	gene	disease		
89,137	418,613(6,839)	276,539(1)	16,215	104

Time measured on NVIDIA Tesla K80 GPU 24GB

## Discussion

Machines are much faster at processing knowledge compared to humans, but they need annotated datasets for training. The overall outcome of the experiments shows that the MGR base dataset can be used to successfully train and evaluate machine-learning models. As far as the dataset size is concerned, Table 3 highlights that the MGR base dataset size is comparable to other datasets for relation extraction, such as the BioCreative V Track 3 datasets.

The task of relation extraction is typically cast as a classification problem, and machine learning methods usually exploit entities mentions co-occurring within a text snippet to decide whether two specific mentions are connected by a relation. On the other hand, typical curation applications for biomedical research only need to know the documents that support the existence of a relation between two given entities. To meet both the points above, the MGR base dataset contains both the concept- and mention-level annotations on the same set of articles.

One of the main problems we had to face to build the annotated dataset was related to entity extraction. In fact, MGDB does not specify the position of the entities in the snippets, and multiple mentions of an entity can occur in a snippet. This could lead to the generation of a number of relations larger than expected (see “MGR base dataset collection” section). However, we observed that many of such potentially problematic relations are actually generated from snippets that contain the acronym of a gene (gene official symbol) in parentheses after the first time the gene official full name is used. Such cases are correct because they represent genuine gene-melanoma relations expressed in the snippet. So, we believe that the truly problematic relations in the produced dataset should constitute a small minority only. In particular cases where a gene is mentioned several times in the same sentence would make it difficult to establish which fragment of the sentence correctly expresses the relationship.

Another issue we had to address was that melanoma disease can be mentioned in texts in many direct and indirect ways. Although this resulted in the loss of a fairly limited number of relations (Table 2), we are currently investigating coreference-resolution techniques for solving those cases of missing relations due to the missing coreference of terms such as tumor or cancer with melanoma.

Regarding the effectiveness of our models, Table 4 compares the results of the models trained on the mention-level training set and evaluated on the concept-level test set with that of the two baselines calculated on the same data. In this context, not only do the models obtain higher *F*_1_ values than the baselines, but they also show a better balance between recall and precision. That is a desirable property for large-scale annotation where the relations extracted from a potentially large number of relations must have a certain degree of accuracy. Interestingly, the produced models get higher accuracy than that of the best systems at BioCreative V Track 3 and at BioCreative VI Track 5, in tasks that are similar, even though not exactly the same. These systems used classical machine learning algorithms and deep learning algorithms. At BioCreative V Track 3, the best system combined two Support Vector Machine (SVM) classifiers. At BioCreative VI Track 5, the best results were obtained by two ensemble systems that combined the results from three models: SVM, CNN and RNN. Finally, BioBERT results are comparable with state-of-the-art results obtained by the BioBERT authors on the BioCreative VI Track 5 dataset [28].

To check the ability of the models to extract exactly the relations that have also been annotated in MGDB, we tested the models on the mention-level test set. Our results (see Table 4, recall values in brackets) have been in line with that of the best system at SemEval-2013 Task 9. The *one snippet per relation* constraint used in MGDB (“MGR base dataset collection” section) is the main reason for not having other evaluation measures (e.g., precision) for this level of annotation. In fact, nominally false positive examples could be produced for those relations that, despite being correctly extracted by models, have not been annotated in the MGR base test set due to the *one snippet per relation* constraint of MGDB. Several of these annotations are in fact perfectly good, but because of this limitation we could not calculate reliable precision values on the mention-level annotation of the dataset.

Turning now to the verification of the models for large-scale data annotation, the figures reported in Table 5 show that the models can extract information with a reasonable speed. An outcome of this data processing has been the annotation of 16,215 relations from 89,137 publications about melanoma. As regards to the quality of these relations, it is worth noting that a high number of publications in the MGR base dataset (891 out of 907) is actually included in the original 90,028 publications abstracts obtained from the initial PubMed query which we used to create the MGR extended dataset. This enables an indirect assessment of the quality of the relations extracted on the basis of the results of the models presented in this study. Among the extracted relations we have found 6,866 new potential gene-melanoma relations related to 2,265 genes not yet classified in MGDB which therefore deserve special attention. In this regard, we have formed a committee of domain experts able to assess the quality of a sample subset of 700 relations (431 genes). The obtained results show that these relations are of good quality (precision: 85.71). 392 out of 431 genes can be assumed to be linked to melanoma (based on the information provided in the original papers) and can be considered for integration with existing structured knowledge databases.

We are confident that our results may improve knowledge about genes that promote melanoma cell growth and proliferation. The MGR base dataset will enable researchers to use complex machine learning algorithms to predict new associations. The thousands of relations extracted from the literature, which are included in the MGR extended dataset, will make it easier for researchers to harvest data from publications. We have implemented a web-based application 9 to quickly and easily browse through the MGR extended dataset.

## Conclusions

This paper describes an annotated dataset of causal relations between genes and melanoma, which contains both concept-level and mention-level annotations. The former is the default format used in curation tasks for biomedical research, such as the BioCreative V Track 3 and BioCreative VI Track 5. The latter is the standard format of annotations used for training text mining tools. We believe that this dataset will be helpful to support the use of machine learning for text mining in the field of melanoma. The trained models enable large-scale and low-cost data annotation. We used them to extract thousands of relations from a large collection of publications about melanoma. Both the annotated dataset and the large number of relations extracted from PubMed have been made available to the research community. We hope that our research will be helpful for facilitating the development of computational approaches for automatic gene-melanoma relation extraction.

## Data Availability

We make both the MGR base dataset and MGR extended dataset freely available in the Mendeley Data repository^11^. The pre-processed data used for training and testing the best model (BioBERT) are also included in the repository.

## Abbreviations

CART: Classification And Regression Tree
CNN: Convolutional Neural Network
BERT: Bidirectional Encoder Representations from Transformers
BioBERT: Bidirectional Encoder Representations from Transformers for Biomedical Text Mining
MeSH: Medical Subject Headings
MGDB: Melanoma Gene Database
MGR: MelanoBase Gene Relation
MIM: Mendelian Inheritance in Man
NLP: Natural Language Processing
OGER: OntoGene Entity Recognition
PMID: PubMed Identifier

## Footnotes

1 http://melanomamine.bioinfo.cnio.es

2 https://public.ndexbio.org/#/user/564dc6ef-8d86-11e5-b435-06603eb7f303

3 https://www.ncbi.nlm.nih.gov/pubmed/

4 https://data.mendeley.com/datasets/745bpf597f/draft?a=ac37c53a-9e50-4ee4-9a96-255d74bb66b0

5 https://spacy.io

6 https://scikit-learn.org

7 http://evexdb.org/pmresources/vec-space-models/PubMed-and-PMC-w2v.bin

8 https://data.mendeley.com/datasets/745bpf597f/draft?a=ac37c53a-9e50-4ee4-9a96-255d74bb66b0

9 https://hltnlp-website.fbk.eu/melanobasedemo/
